# Contamination and risk assessment of heavy metals, and uranium of sediments in two watersheds in Abiete-Toko gold district, Southern Cameroon

**DOI:** 10.1016/j.heliyon.2019.e02591

**Published:** 2019-10-05

**Authors:** Eugène Pascal Binam Mandeng, Louise Marie Bondjè Bidjeck, Armel Zacharie Ekoa Bessa, Yvan Demonstel Ntomb, Jacques Wassouo Wadjou, Elvine Paternie Edjengte Doumo, Lucien Bitom Dieudonné

**Affiliations:** a Centre for Geological and Mining Research, Garoua, Cameroon; b Department of Earth Sciences, University of Yaoundé I, Yaoundé, Cameroon; c Department of Earth Sciences, Faculty of Science, University of Ngaoundere, Cameroon; d Faculté d’Agronomie et des Sciences Agricoles, University of Dschang, Cameroon

**Keywords:** Applied ecology, Soil pollution, Environmental assessment, Waste treatment, Environmental impact assessment, Environmental radioactivity, Environmental risk assessment, Abiete-toko watersheds, Enrichment factor, Geo-accumulation, Ecological risk assessment, Contamination, Sediments

## Abstract

In this investigation, the level of toxic metals (Cd, Pb, Hg, Cu, Ni, Al, Zn and U) was determined in sediment samples from two watersheds (Kienké and Tchangué) in the Abiete-Toko gold district, southern Cameroon. The potential contamination and toxicity of studied metals was determined by evaluating enrichment factor (EF), geo-accumulation index (Igeo) and ecological risk assessment (ERA). Considering the spatial distribution patterns, metal concentrations were lower than the average shale values, except for Cu and Ni of site 4 in the Kienké watershed and only Ni in the Tchangué watershed. In this study, the EF and Igeo values revealed that sediments were moderately polluted by Ni and Cu and unpolluted by other metals. The evaluation of the ERA based on ecological risk index (RI), ecological risk factor (Er), contamination factor (CF) and pollution load index (PLI) revealed that the sediments from the Abiete-Toko watersheds have significant to very high ecological risk assessment and are generally unpolluted by trace metals and U, except for Ni and Cu. Little quantities of heavy metals with low U levels and distribution were found at the sites close to the vicinity of artisanal mining and peri-urban areas. This proximity reveals that artisanal gold mining activities, agricultural runoff, and other anthropogenic inputs in the study area are probable sources of slight metal contamination. However, the non-use of toxic effluents for gold mining and pesticides for agriculture can be an advantage of the unpolluted status of the watersheds. The physical degradation of the ecosystem through excavations, wells and other stream diversion methods is expanding in the zone. Appropriate measures should be taken by artisans to rehabilitate the gold mining sites, to ensure appropriate treatment of wastewater and non-use of toxic effluents into nearby tributaries.

## 1 Introduction

Contamination by trace metals is a serious threat in aquatic systems due to their level of toxicity, abundance, persistence in the environment and subsequent accumulation in aquatic milieu (Bessa et al., 2018; Ekoa Bessa et al., 2018). In some studies, it was reported that the contamination of flooded areas by trace elements includes natural and anthropogenic sources such as geological weathering and erosion (Kaushik et al., 2009; Hanif et al., 2016), atmospheric deposition (Huang et al., 2009; Xiong et al., 2016; Ekoa Bessa et al., 2018), disposal of liquid effluents, fertilizers, and pesticides (Sun et al., 2018; Zhou et al., 2018; Jiang et al., 2019), terrestrial run-off and chemicals originating from various urban, industrial and agricultural activities (Xiao et al., 2013; Zahra et al., 2014; Mimba et al., 2018; Al-Hadithy et al., 2018; Chen et al., 2019). Sediments are the source of substrate nutrients, micro- and macro flora and fauna have a significant role in the aquatic habitat (Jain et al., 2004; Guo et al., 2010; Bat and Özkan, 2019; Gao et al., 2019). Nevertheless, some studies have suggested that sediments could serve as an indicator for contamination levels and could act as a screening tool to fingerprint of environmental pollution in the surrounding environment (Xiao et al., 2013; Zahra et al., 2014; Ekoa Bessa et al., 2018). The high levels of trace metals in aquatic sediments may pose a potential risk to human health due to their transfer into marine biota, and eventually into food chain (Varol and Şen, 2012; Dessalew et al., 2018; Bhattacharyya et al., 2019).

Natural radiation has permanently been part of the human environment. Its principal mechanisms are cosmogenic radiation and cosmic, terrestrial gamma radiation from natural radionuclides in rocks, soils and sediments and natural radioactive substances in our diet and inhaled air (Bochicchio et al., 1995; Lu and Liu, 2018; Bartlett and Castro, 2019). Radionuclides are found as naturally occurring elements and as products or by-products of nuclear technologies. One of the most common radionuclides is uranium (U). As all isotopes of uranium are radioactive, it is very important to control their quantity (Van Nostrand et al., 2009; Xiang et al., 2017; Wang et al., 2017). Nuclear fission in connection with atomic weapons testing and nuclear power generation provides some of the sources of sediment and water contamination, the major part of radionuclides released into the environment will finally accumulate in either the upper layer of soils or interstitial system of sediments in aquatic systems (Igwe et al., 2005; Carvalho et al., 2014; Xiang et al., 2016; Ma et al., 2019). Once the uranium enters in the organism, it is transferred to extracellular fluids and transported through the blood to other organs. The risks related to exposure to uranium can be both chemical and radiological, which can be related to the binding of U to biological molecules (Xiang et al., 2017; Lu and Liu, 2018). In Abiete-Toko, some explorers noticed the presence of heavy metals and uranium, and warned the populations on the effect of heavy metals and uranium radiation, which inspired the authors to conduct this study.

The main aim of the present study is to evaluate the levels of trace metals and U in the sediments from the Abiete-Toko watersheds in order to identify their naturally enriched or anthropogenic sources using heavy metal indices as well as to assess the environmental risk of heavy metal in the investigated area.

## 2 Materials & methods

### 2.1 Study area

**Fig. 1:**
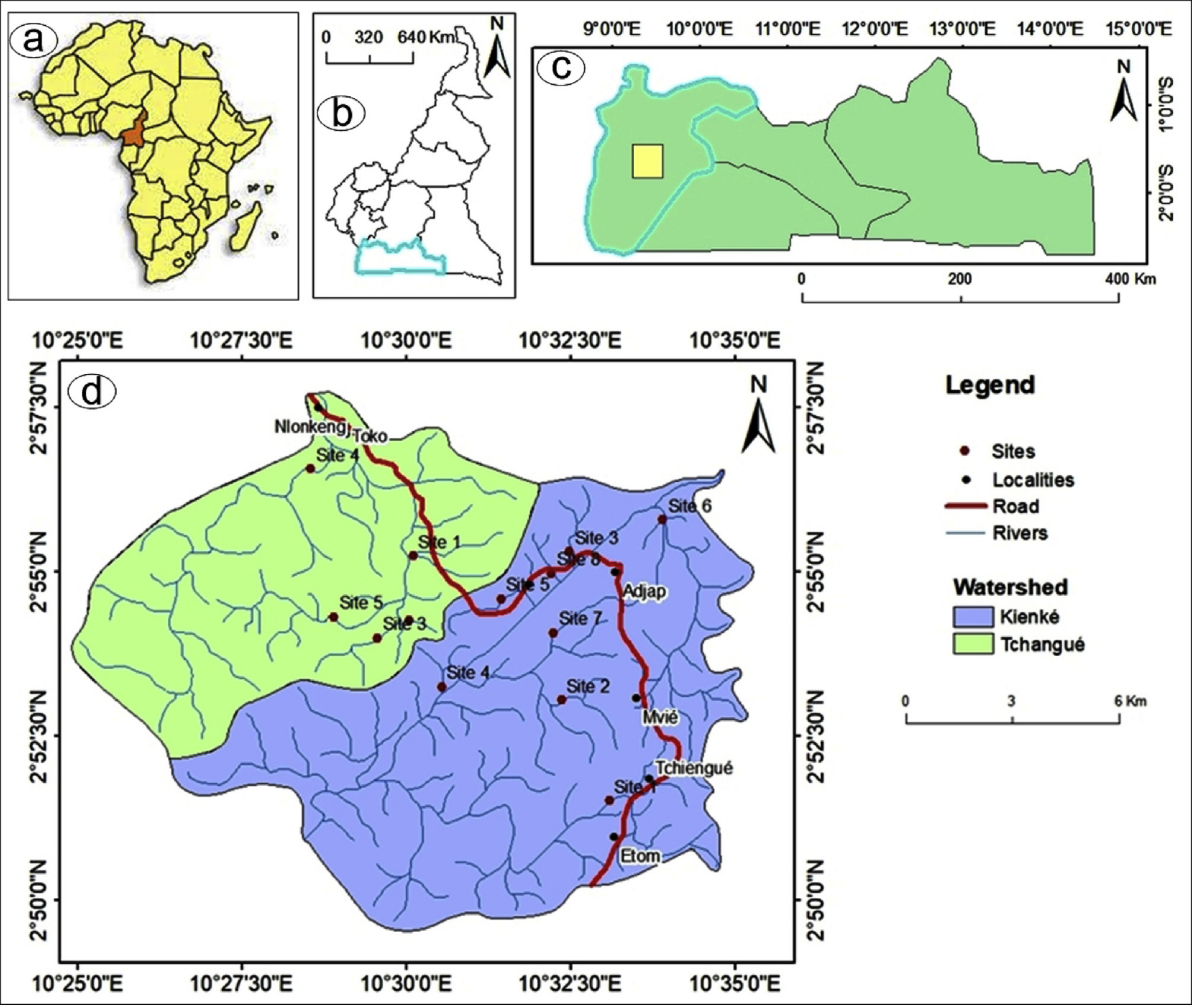
Investigated area and sampling sites inside the Abiete-Toko gold district: a), b) and c). Location of the study area; d) Kienké and Tchangué watersheds.

The studied watersheds are located in Abiete and Toko villages which are part of the Akom II District in Southern-Cameroon. The first watershed called the Kienké watershed is located in Abiete and the second called Toko is located in the Tchangué watershed. They have a dendritic hydrographic network (Fig. 1). The current equatorial type climate, characterized by an average precipitation climatically constrained to 1800 mm/yr, and an average temperature of 24.8 °C favours the development of a dense forest of equatorial type (Letouzey, 1985) and the development of ferrallitic soils on the interfluves and hydromorphic soils downstream (Bilong et al., 1992). These make surface observation and mapping fairly challenging.

Geologically, the study area is located within the north-western border of the Congo Craton called the Ntem complex (composed of the Nyong Unit at the West, the Ntem unit at the centre and the Ayina Unit at the East) with ages ranging from 2400–1800Ma complex in Cameroon (Maurizot et al., 1986; Shang et al., 2007; Sylvestre et al., 2017). The Nyong unit in which the Abiete-Toko gold district is located is mainly made up of tonalitic to granitic gneisses associated with amphibolites, greenstone belts, syntectonic plutonism and metasedimentary rocks recrystallized under high-grade metamorphic conditions at ca. 2050 Ma (Nédélec et al., 1993; Toteu et al., 2004; Lerouge et al., 2006; Binam Mandeng et al., 2018). Locally, the site is based on a set of mafic and ultra-mafic rocks including gabbro, amphibolites, pyroxenites and peridotites associated with gneiss and quartzite (Ngo Bidjeck, 2004; Binam Mandeng et al., 2018). Small-scale gold mining is the major industrial activity in the area. To date, no study has proposed an assessment of the potential risks associated with this activity and over 80% of the inhabitants are subsistent farmers and depend on surface water and groundwater for domestic purposes.

### 2.2 Sampling and analysis of sediments

In August 2018, a total of 13 composite sediment samples were collected from selected rivers of the Abiete-Toko watersheds (8 in the Kienké watershed at Abiete and 5 in the Tchangué watershed at Toko) and at each point, composite sediment samples were collected at the top (0–5 cm) of sediment deposits using a Peterson mud sampler (XDB0201). At each sampling site, 5 kg of surface sediment sample was selected and stored in closed plastic packaging bags. In addition, the geographical location of each sample site was gotten using a GPS. Sediment samples were air-dried. Stones, rubbles, and plant roots removed manually. After grinding with an agate mortar, soil samples were sieved using the 0.080 mm sieve to remove small debris. These sieved sediment samples were stored in closed plastic bags for further analysis. Pre-treatment of Cd, Pb, Hg, Cu, Ni, Al, Zn and U of sediment samples was determined with reference to ‘methods for chemical analysis of silicate rocks’ (GB/T 14506.30–2010). The concentration of each element was analysed by inductively coupled plasma mass spectrometer (ICP-MS) from the ALS Global Group (Vancouver, Canada). For these samples, the adding standard recovery test of the determined elements was performed and the adding recovery rate of the samples were observed within 82.3–103%. The relative standard deviation (RSD) range is between 2.3 and 12% (parallel determination six times).

### 2.3 Heavy metals indices

#### 2.3.1 Enrichment factor (EF)

The enrichment factor (EF) is generally used as an appropriate method to discriminate between natural and anthropogenic sources and to reflect the status of environmental contamination, based on the use of a normalization element in order to improve the variations produced by heterogeneous sediments (Zahra et al., 2014; Hanif et al., 2016). Metal concentrations were normalized in the sediments with respect to Al, used as reference material. According to Salati and Moore (2010), the EF of metals/metalloids in the sediments at all the stations was calculated as follows:

*EF = (M /Al) sample / (M / Al) background*

where *M sample* and *M background* are the contents of the investigated metals (Cd, Pb, Hg, Cu, Ni, Al, Zn and U) in the sediment samples and uncontaminated background respectively; and *Al sample* and *Al background* are the contents of Al in sediment samples and uncontaminated background respectively. In this study, baseline values for M background and Al background were adopted from Turekian and Wedepohl (1961). The EF values were interpreted as reported in Table 1.

**Table 1:** Classes of EF, I-geo, Er, RI and CF in relation to enrichment, pollution, potential ecological risk, ecological risk and contamination levels, respectively.

EF classes	Enrichment level	I-geo value; classes	Pollution level
EF < 1	No enrichment	I-geo ≤ 0; 0	Unpolluted
EF = 1-3	Minor enrichment	I-geo = 0–1; 1	Unpolluted to moderately polluted
EF = 3-5	Moderate enrichment	I-geo = 1–2; 2	Moderately polluted
EF = 5-10	Moderately severe enrichment	I-geo = 2–3; 3	Moderately to strongly polluted
EF = 25-50	Very severe enrichment	I-geo = 3–4; 4	Strongly polluted
EF > 50	Extremely severe enrichment	I-geo = 4–5; 5	Strongly to very strongly polluted

RI classes	Risk level	CF classes	Contamination level

RI < 150	Low ecological risk	CF < 1	Low contamination
RI = 150-300	Moderate ecological risk	CF = 1-3	Moderate contamination
RI = 300-600	Significant ecological risk	CF = 3-6	Considerable contamination
RI > 600	High ecological risk	CF > 6	High contamination

Er classes	Er level		

Er < 40	Low potential ecological risk		
Er = 40-80	Moderate potential ecological risk		
Er = 80-160	Significant potential ecological risk		
Er = 160-320	High potential ecological risk		
Er > 320	Very high potential ecological risk		

#### 2.3.2 Geo-accumulation index (I*geo*)

The geo-accumulation index (Igeo) for the metal/metalloids concentrations was calculated using the following formula (Muller, 1969):

*Igeo = Log2 (C sample / 1.5 B sample)*

where *C sample* is the measured concentration (mg/kg) of metal in the sample sediment, *B sample* is the geochemical background value (mg/kg) of the element in the background sample and the factor *1.5* is introduced to minimize the effects of possible variations in the background values which may be attributed to lithogenic effects. The background data used is from Turekian and Wedepohl (1961). Geo-accumulation Index values were interpreted as reported in Table 1.

#### 2.3.3 Ecological risk assessment (ERA)

The ERA was carried out by potential ecological risk index (RI) for this study. The potential ecological risk index (RI) of the heavy metals is known as the sum of the risk factors, and it has been developed for six toxic metals using the equations of Hakanson (1980) and Zhu et al. (2008).

$$RI=\overset{n}{\underset{1}{\sum }}Er\text{ ​and ​}Er=Tr\;\times CF$$

where *Er* is the single index of ecological risk factor, and n is the amount of the heavy metal class, *Tr* = toxic response factor suggested by Hakanson (1980) for six metals Cd (10), Pb (5), Hg (40), Cu (5), Ni (5), Zn (1). *Er* and *RI* express the potential ecological risk factor of individual and multiple metals respectively. The expressions and values used for the interpretation of the potential ecological risk factor (Hakanson, 1980) are reported in Table 1.

The pollution load index (PLI) of a single site is the root number (*n*) of multiplied together contamination factor (CF) values.

$$PLI=\sqrt[{n}]{(CF1\times CF2\times CF3\times \ldots \ldots \times CFn}$$

where, *n* is the number of metals and *CF* is the contamination factor. The pollution load index was interpreted by Harikumar et al. (2009). A PLI value under zero indicates unpolluted soils or sediments; zero indicates perfection; a value of one indicates the presence of only baseline levels of pollutants and values above one would indicate progressive deterioration of the site quality (Tomilnson et al., 1980; Seshan et al., 2010).

## 3 Results and discussion

### 3.1 Occurrence and distribution of trace metals in the sediments of the Abiete-Toko watersheds

**Fig. 2:**
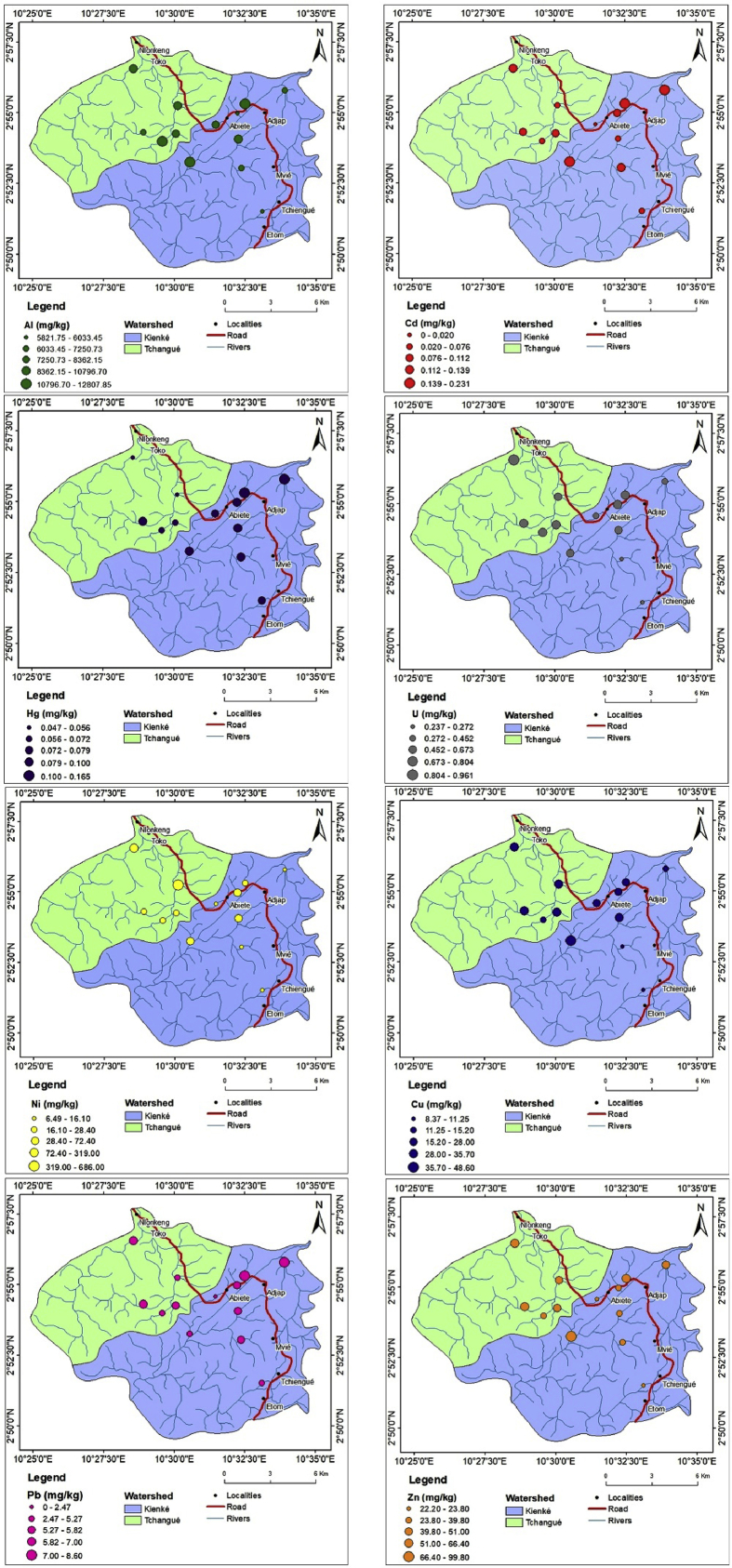
Distribution maps of the concentration of selected heavy metals (mg/kg) and U in sediments of the Abiete-Toko watersheds.

The basic descriptive statistical values and spatial distribution patterns of the studied trace metals and uranium are presented in Table 2 and Fig. 2. On average basis, the metals follow a decreasing concentration order Al > Zn > Ni > Cu > Pb > U > Cd > Hg in the Kienké watershed (Abiete) and Al > Ni > Zn > Cu > Pb > U > Cd > Hg in the Tchangué watershed (Toko). Comparing the average concentrations of heavy metals in the different sites and samples, it is noticed that the average Al concentration is higher (9011.48 mg/kg) than that of other metals. This Al average concentration in the Abiete-Toko sediments is below the Average Shale (80000 mg/kg) (Turekian and Wedepohl, 1961) reference values. On the other hand, the average concentrations of the other metals in this study are less than the reference values of Average Shale (Turekian and Wedepohl, 1961) and EU (2002) values, except the values of Ni (reference values of Average Shale ≈68 mg/kg after Turekian and Wedepohl, 1961) in site 7 in the Kienké watershed (72.4 mg/kg) and sites 1 and 4 in the Tchangué watershed (686 and 217.28 mg/kg respectively). These abnormal values can be attributed to the result of natural weathering and leaching of rocks.

**Table 2:** Heavy metal and Uranium concentrations in mg/kg (mean of two replicates) in surface sediment (0–5 cm) in The Abiete-Toko watersheds.

	Site	Al	Cd	Pb	Hg	Cu	Ni	Zn	U
Kienké watershed (Abiete)	Site 1	5821.75	0.075	5.26	0.079	8.37	6.69	22.2	0.272
	Site 2	6615.63	0.131	5.8	0.098	11.25	9.29	36.5	0.237
	Site 3	12013.98	0.22	8.6	0.165	28	28.4	64	0.778
	Site 4	12807.85	0.193	5.17	0.093	48.6	66.1	99.8	0.673
	Site 5	8362.15	0.02	2.47	0.077	24	9.15	23.8	0.381
	Site 6	7091.95	0.231	8.22	0.145	13.25	27.1	51	0.452
	Site 7	9261.875	0.057	5.67	0.1	35.7	72.4	36.2	0.673
	Site 8	6033.45	0.095	5.66	0.091	24.4	59	39.8	0.804
	Mean	8501.08	0.128	5.856	0.106	24.196	34.77	46.662	0.534
Tchangué watershed (Toko)	Site 1	10796.7	0.068	5.27	0.056	30	686	47.8	0.613
	Site 2	8309.23	0.099	5.82	0.068	32.2	26.1	46.8	0.751
	Site 3	12119.83	0.076	5.24	0.072	15.2	27.1	32.3	0.78
	Site 4	9473.58	0.139	6.55	0.047	31.7	319	66.4	0.961
	Site 5	7250.73	0.112	7	0.097	29.9	28.2	58.8	0.799
	Mean	9590.01	0.099	5.976	0.068	27.8	217.28	50.42	0.781
Average shale∗		80000	0.3	20	0.4	45	68	95	3.7
EU (2002)		-	0.3	300	1.1	40	75	300	-

EU: European Union Standards.

∗ Turekian and Wedepohl (1961).

Aluminium is the third most abundant element in the earth's crust. It is naturally present in our environment: sediments, water and food. Its presence is of concern because it is suspected of increasing the risk of dementia or certain cancers (Malik et al., 2004; Fashola et al., 2016). In the present study, the concentration in aluminium was higher than other metals in both watersheds, indicating that this metal is naturally high in the sediments. However, the concentrations of aluminium were higher in the Tchangué watershed than in the Kienké. The range of aluminium in the present results also is less than those recorded by Silveira et al. (2016) in sediments from Piabanha watershed in Brasil, and Bessa et al. (2018) in Simbock Lake sediments.

Nickel is a widespread metal/metalloid in the environment. Its sources can be: electroplating, non-ferrous metal, paints and porcelain enamelling. The effects of Ni in the human organism are cardiovascular diseases, chest pain, dermatitis, dizziness, dry cough and shortness of breath, headache, kidney diseases, lung and nasal cancer and nausea (Malik et al., 2004; Fashola et al., 2016). The highest value of Ni in the Kienké watershed was observed at site 7. In the Tchangué watershed, its highest value was recorded in sites 1 and 4. Those maxima were recorded near artisanal gold mining sites. The highest Ni concentrations could be ascribed to its accumulation at the surface of sediments from deposition by artisanal gold mining and agricultural activities (Cempel and Nikel, 2006). In this precise context, it could also be associated with the alteration of ultramafic rocks such as peridotite. Nickel values from this study are higher than the reference values of Average Shale (Turekian and Wedepohl, 1961) and EU (2002) values. The same values were observed in Moloundou swamp sediments (Ekoa Bessa et al., 2018) and sediments from the Rhumel and Sakiet Roum wadis, in the industrial zone in Constantine, Algeria (Afri-Mehennaoui et al., 2009).

Copper and Zinc are few represented in the study; their average concentrations are generally below the Average Shale (Turekian and Wedepohl, 1961) and EU (2002) values, except for site 4 in the Kienké watershed which is near an artisanal gold exploitation site, where highest concentrations are recorded (48.6 and 99.8 mg/kg for copper and zinc respectively). In the Kienké watershed, their mean values are 24.19 mg/kg (Cu) and 46.7 mg/kg (Zn) while in the Tchangué watershed their mean values are 27.8 mg/kg (Cu) and 50.42 mg/kg (Zn). These concentrations are lower than those reported for the sediments of Tigris river, Turkey (Varol and Şen, 2012), Indian rivers (Singh et al., 2005; Suthar et al., 2009), Second Songhua river, China (Lin et al., 2008) and Simbock Lake in Mefou river, Cameroon (Bessa et al., 2018). High doses or even an overdose of copper and zinc can cause important side effects: Ataxia, depression, gastrointestinal irritation, haematuria, icterus, impotence, kidney and liver failure, lethargy, macular degeneration, metal fume fever, prostate cancer, seizures and vomiting for Zinc (Gumpu et al., 2015), and abdominal pain, anaemia, diarrhoea, headache, liver and kidney damage, metabolic disorders, nausea and vomiting for copper (Salem et al., 2000; Fashola et al., 2016). Copper and zinc can have multivariable sources such as brass manufacturing, mining, oil refinery and plumbing. From this study, the presence of Cu and Zn could be due to fragmentation of rocks in the area and from artisanal mining activities.

Lead is the most immobile element and its content in sediment is closely associated with clayminerals (El-Alfy et al., 2017). It can be the cause of many negative effects on humans such as anorexia, chronic nephropathy, damage to neurons, high blood pressure, hyperactivity, insomnia, learning deficits, reduced fertility, renal system damage, risk factor for Alzheimer's disease and shortened attention span. Apart from natural origin, Pb has numerous sources: coal combustion, electroplating, manufacturing of batteries, mining, paint and pigments (Ali et al., 2013; Fashola et al., 2016). In this study, mean concentrations of Pb are 5.86 and 5.98 mg/kg in the Kienké and Tchangué watersheds respectively. These concentrations are below the Average Shale (Turekian and Wedepohl, 1961), and lower than those reported for sediments of the Nile delta, Egypt (El-Alfy et al., 2017); Chenab River, Pakistan (Hanif et al., 2016); Ganga river, India (Sinha and Paul, 2015) and Simbock Lake in Mefou river, Cameroon (Bessa et al., 2018). The low range of Pb can be attributed to the incorporation of lead in the leaching of source rocks and agricultural soil through various types of pollutions (Alloway, 1990; Ekoa Bessa et al., 2018).

Cadmium is a metal that occurs naturally at low levels in the environment (Stankovic, 2011). It can also be the result of fertilizer, mining, pesticide, plastic, refining and welding which can have effects on humans such as bone disease, coughing, emphysema, headache, hypertension, kidney diseases, lung and prostate cancer, lymphocytosis, microcytic hypochromic anemia, testicular atrophy and vomiting (Sebogodi and Babalola, 2011; Fashola et al., 2016). The mean range of Cd in studied locations was 0.13 and 0.1 mg/kg for the Kienké and Tchangué watersheds respectively. These values are lower than the Average Shale (Turekian and Wedepohl, 1961), and those reported for sediments of Rimac river, Peru (Méndez, 2005); Tigris river, Turkey (Varol and Şen, 2012) and from the paddy rice fields of Pakistan (Abdullah et al., 2015). Low concentrations of Cd can be attributed to low leaching of source rocks.

Mercury is one of the most toxic heavy metals that can cause pollution. This is essentially due to the gas phase which will allow the different transfers in space and with matter. Batteries, coal combustion, geothermal activities, mining, paint industries, paper industry, volcanic eruption and weathering of rocks are sources of Hg. They can have some effects on human such as: ataxia, attention deficit, blindness, deafness, decrease rate of fertility, dementia, dizziness, dysphasia, gastrointestinal irritation, gingivitis, kidney problem, loss of memory, pulmonary edema, reduced immunity and sclerosis (Wang et al., 2017; Ali et al., 2013; Fashola et al., 2016). In the current study, it is reported that the mean concentrations of Hg (mg/kg) in the sediments are 0.1 and 0.07 in the Kienké and Tchangué watersheds respectively. The comparison between Hg concentrations in the sediments of the Abiete-Toko rivers and those determined in other sites showed that Hg levels in the Kienké and Tchangué watersheds sediments had lower levels than those measured in the sediments of the Mar Piccolo of Taranto, southern Italy (Bellucci et al., 2016), Ganga river, India (Sinha et al., 2007, Sinha and Paul, 2015) and also Average Shale (Turekian and Wedepohl, 1961) and EU (2002) values. Mercury values are also closed to those of agricultural soil around the uranium tailing reservoir in Southern China (Lu and Liu, 2018). The low range of Hg in this study may attributed to the weathering of rocks.

The radioactive metal U is one of the heaviest, naturally-occurring element on earth. With a global background concentration in the earth's crust of approximately 2–4 mg/kg, natural U is approximately 1 000 times more common than gold and 10 times more abundant than other (also toxic) heavy metals such as cadmium (0.3 mg/kg) or mercury (0.4 mg/kg) (Turekian and Wedepohl, 1961). Uranium has biologically dynamic toxicity, metabolic toxicity and chemical toxicity, leading to potential long-term harm to mammalian reproduction and development with reduced biological fertility, abnormal and slow embryonic development (Domingo, 2001). Rock types with an elevated U background include shales (3.7 mg/kg) (Turekian and Wedepohl, 1961). Concerning U in this study, the mean concentrations were 0.53 and 0.78 mg/kg in the Kienké and Tchangué watersheds sediments respectively. Although these values are lower than those reported for sediments in gold mining areas of Witwatersrand (Winde and Sandham, 2004); the Vale De Abrutiga uranium Mine, Central Portugal (Pinto et al., 2004); the agricultural soil around the uranium tailing reservoir in Southern China (Lu and Liu, 2018) and the Ploučnice River, Czech Republic (Grygar et al., 2014). However, in this study, similar values of U were found in south eastern Brazil mine (Abessa et al., 2014). According to the WHO (2011), the mean U concentration in Abiete-Toko river sediments are under the agricultural, residential and industrial limits for environmental health.

### 3.2 Estimation of pollutant indicators

#### 3.2.1 Enrichment factor (EF) and geo-accumulation index (I*geo*)

**Fig. 3:**
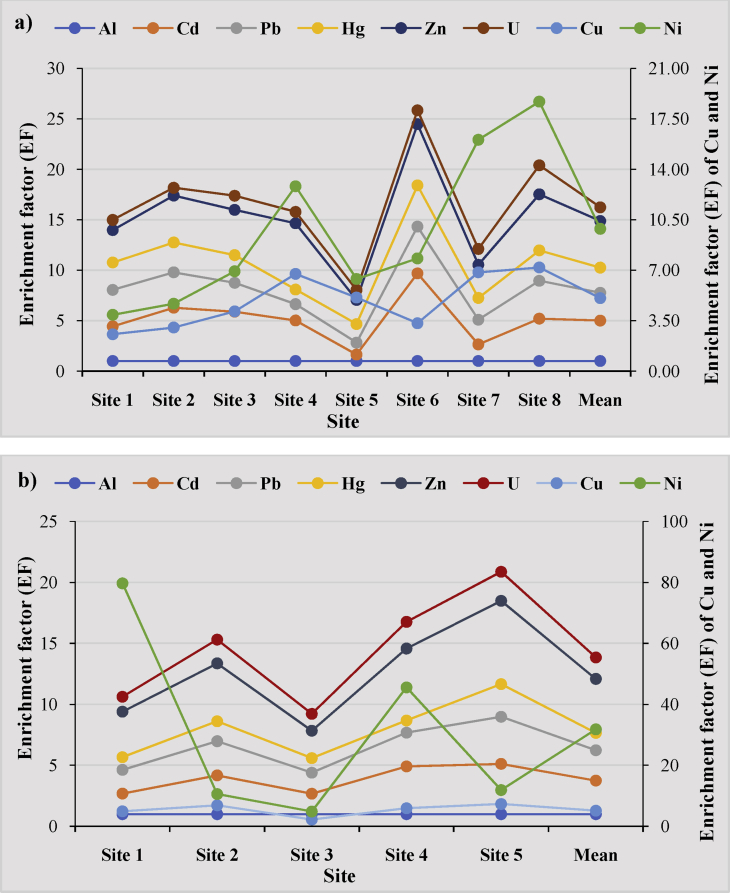
The enrichment factor of heavy metals in the sediments of the Abiete-Toko gold district: a) Kienké watershed; b) Tchangué watershed.

The enrichment factors of heavy metals and U in the Kienké and Tchangué watersheds are shown in Table 3 and Fig. 3a, b. The sequence of EF in the Kienké watershed was U > Hg > Pb > Cd > Zn > Ni > Cu. On the other hand, the sequence of the EF in the Tchangué watershed was as follows; Hg > U > Pb > Cd > Zn > Cu > Ni. The enrichment factor of heavy metals in Abiete-Toko rivers is >2, indicated that the source of these metals was from anthropogenic activities. In this study, it is obvious that Ni is the most abundant in the study area. Its highest value is located in the Tchangué watershed with a very severe enrichment (range value = 25–50). In the two watersheds, Cu has the second most abundant enrichment level with a moderately severe enrichment (range value = 5–10) and the other elements (U, Hg, Pb, Cd, Zn) have minor to moderate enrichment (Table 3 and Fig. 3a, b). This could be attributed to artisanal gold mining and agricultural activities.

**Table 3:** Enrichment factor (EF) for heavy metals in sediments of Abiete-Toko watersheds.

	Site	Al	Cd	Pb	Hg	Cu	Ni	Zn	U
Kienké watershed (Abiete)	Site 1	1	3.44	3.61	2.71	2.56	1.35	3.21	1.01
	Site 2	1	5.28	3.51	2.96	3.02	1.65	4.65	0.77
	Site 3	1	4.88	2.86	2.75	4.14	2.78	4.49	1.40
	Site 4	1	4.02	1.61	1.45	6.75	6.07	6.56	1.14
	Site 5	1	0.64	1.18	1.84	5.10	1.29	2.40	0.99
	Site 6	1	8.69	4.64	4.09	3.32	4.50	6.06	1.38
	Site 7	1	1.64	2.45	2.16	6.85	9.20	3.29	1.57
	Site 8	1	4.20	3.75	3.02	7.19	11.50	5.55	2.88
	Mean	1	4.01	2.76	2.49	5.06	4.81	4.62	1.36
Tchangué watershed (Toko)	Site 1	1	1.68	1.95	1.04	4.94	74.75	3.73	1.23
	Site 2	1	3.18	2.80	1.64	6.89	3.70	4.74	1.95
	Site 3	1	1.67	1.73	1.19	2.23	2.63	2.24	1.39
	Site 4	1	3.91	2.77	0.99	5.95	39.61	5.90	2.19
	Site 5	1	4.12	3.86	2.68	7.33	4.58	6.83	2.38
	Mean	1	2.75	2.49	1.42	5.15	26.66	4.43	1.76

The geo-accumulation index of heavy metals and U in both watersheds of Abiete-Toko showed that the sediments were not polluted (Table 4), they fall in class 0. This could be attributed to the non-use of fertilizers in agricultural activities and toxic products in artisanal gold mining.

**Table 4:** Geoaccumulation index (I-geo) of heavy metals for sediments of selected sites of the Abiete-Toko watersheds.

	Site	Al	Cd	Pb	Hg	Cu	Ni	Zn	U
Kienké watershed (Abiete)	Site 1	-1.31	-6.20	-4.36	-6.18	-4.16	-4.25	-3.73	-5.64
	Site 2	-1.26	-5.96	-4.32	-6.09	-4.03	-4.11	-3.52	-5.70
	Site 3	-1.00	-5.74	-4.14	-5.86	-3.63	-3.63	-3.27	-5.19
	Site 4	-0.97	-5.79	-4.37	-6.11	-3.39	-3.26	-3.08	-5.25
	Site 5	-1.16	-6.78	-4.69	-6.19	-3.70	-4.12	-3.70	-5.50
	Site 6	-1.23	-5.72	-4.16	-5.92	-3.96	-3.65	-3.37	-5.42
	Site 7	-1.11	-6.32	-4.33	-6.08	-3.53	-3.22	-3.52	-5.25
	Site 8	-1.30	-6.10	-4.33	-6.12	-3.69	-3.31	-3.48	-5.17
	Mean	-1.15	-5.97	-4.31	-6.05	-3.70	-3.54	-3.41	-5.35
Tchangué watershed (Toko)	Site 1	-1.05	-6.25	-4.36	-6.33	-3.60	-2.24	-3.40	-5.29
	Site 2	-1.16	-6.08	-4.31	-6.25	-3.57	-3.66	-3.41	-5.20
	Site 3	-1.00	-6.20	-4.36	-6.22	-3.90	-3.65	-3.57	-5.19
	Site 4	-1.10	-5.94	-4.26	-6.41	-3.58	-2.58	-3.26	-5.10
	Site 5	-1.22	-6.03	-4.23	-6.09	-3.60	-3.63	-3.31	-5.18
	Mean	-1.10	-6.08	-4.30	-6.25	-3.64	-2.74	-3.38	-5.19

#### 3.2.2 Ecological risk assessment

##### 3.2.2.1 The ecological risk factor (Er) and the ecological risk index (RI)

**Fig. 4:**
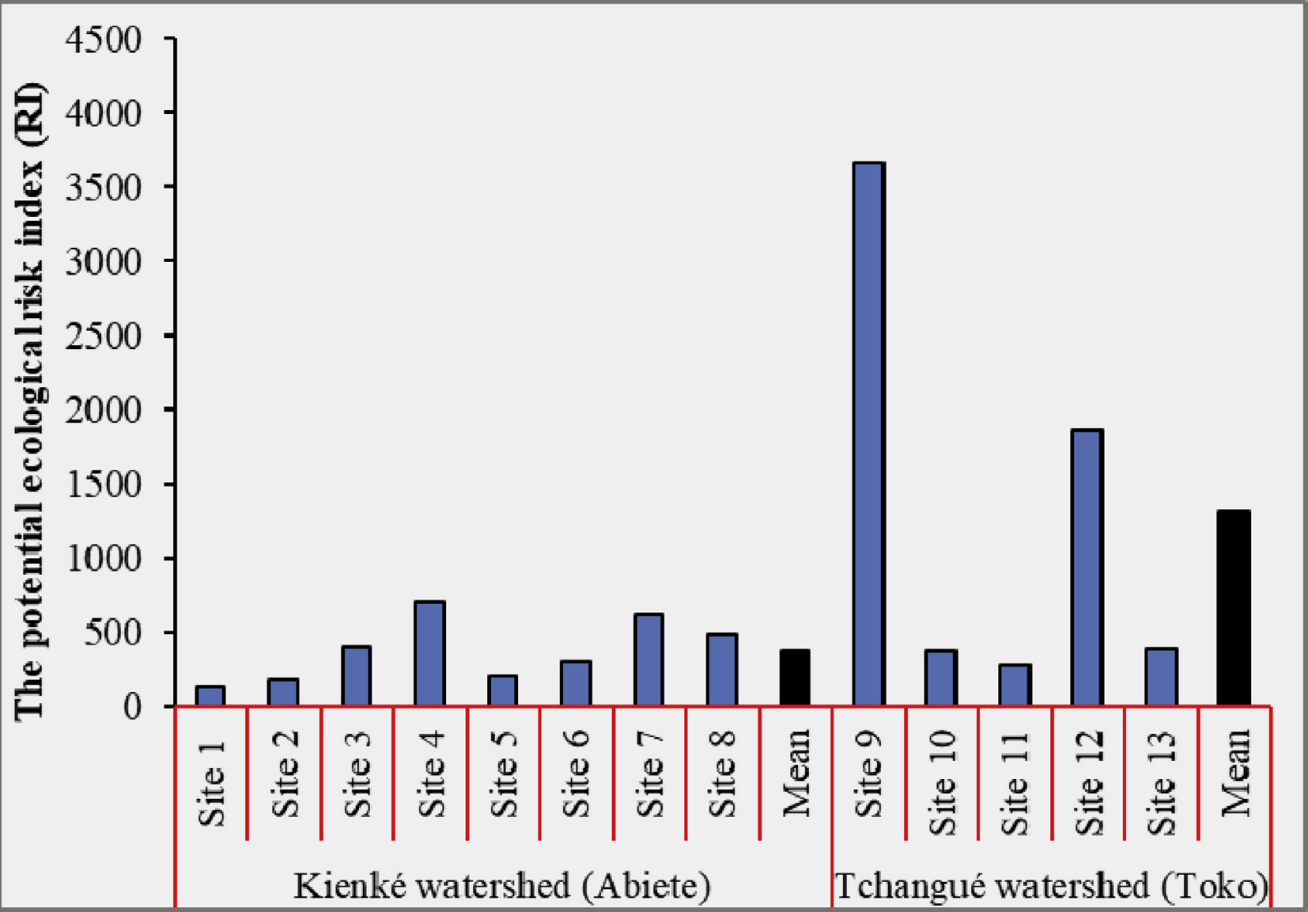
The ecological risk factor (Er) and the ecological risk index (RI) of heavy metals in the sediments of the Abiete-Toko gold district: a) Kienké watershed; b) Tchangué watershed.

The Er and RI of heavy metals in the investigated sites in the two watersheds of the Abiete-Toko gold district are given in Table 5 and Fig. 4a, b.

**Table 5:** Pollution indices (Er and RI) in the sediment of the Abiete-Toko watersheds.

	Site	Ecological risk factor (Er)						RI
		Cd	Pb	Hg	Cu	Ni	Zn	
Kienké watershed (Abiete)	Site 1	2.25	26.3	3.16	41.85	33.45	22.2	129.21
	Site 2	3.93	29	3.92	56.25	46.45	36.5	176.05
	Site 3	6.6	43	6.6	140	142	64	402.2
	Site 4	5.79	25.85	3.72	243	330.5	99.8	708.66
	Site 5	0.6	12.35	3.08	120	45.75	23.8	205.58
	Site 6	6.93	41.1	5.8	66.25	135.5	51	306.58
	Site 7	1.71	28.35	4	178.5	362	36.2	610.76
	Site 8	2.85	28.3	3.64	122	295	39.8	491.59
	Mean	3.83	29.28	4.24	120.98	173.83	46.66	378.83
Tchangué watershed (Toko)	Site 1	2.04	26.35	2.24	150	3430	47.8	3658.43
	Site 2	2.97	29.1	2.72	161	130.5	46.8	373.09
	Site 3	2.28	26.2	2.88	76	135.5	32.3	275.16
	Site 4	4.17	32.75	1.88	158.5	1595	66.4	1858.7
	Site 5	3.36	35	3.88	149.5	141	58.8	391.54
	Mean	2.964	29.88	2.72	139	1086.4	50.42	1311.38

RI: Potential ecological risk index.

The Er in the Kienké and Tchangué watersheds showed low potential ecological risk factor (Er < 40) for the trio Cd, Pb and Hg; Zn and Cu have moderate and significant potential ecological risk factor respectively. Nickel has the highest Er level in all selected rivers of the watersheds. In the Kienké watershed, it ranges between 160-320 with a high potential ecological risk and in the Tchangué watershed, the level of potential ecological risk is very high with values reaching 320.

The RI of the studied trace metals in sediments in the Abiete-Toko watersheds showed significant and high ecological risk index. In Kienké rivers, mean value of RI range is considered to be at risk of contamination with a significant ecological risk index, except in site 4 where the indices are a high ecological risk. The same contamination is seen in the Tchangué watershed with a high ecological risk (mean ≈1311.38). This indicates high risk of contamination according to Adebowale et al. (2009) and Ekoa Bessa et al. (2018).

##### 3.2.2.2 The contamination factor (CF) and pollution load index (PLI)

**Fig. 5:**
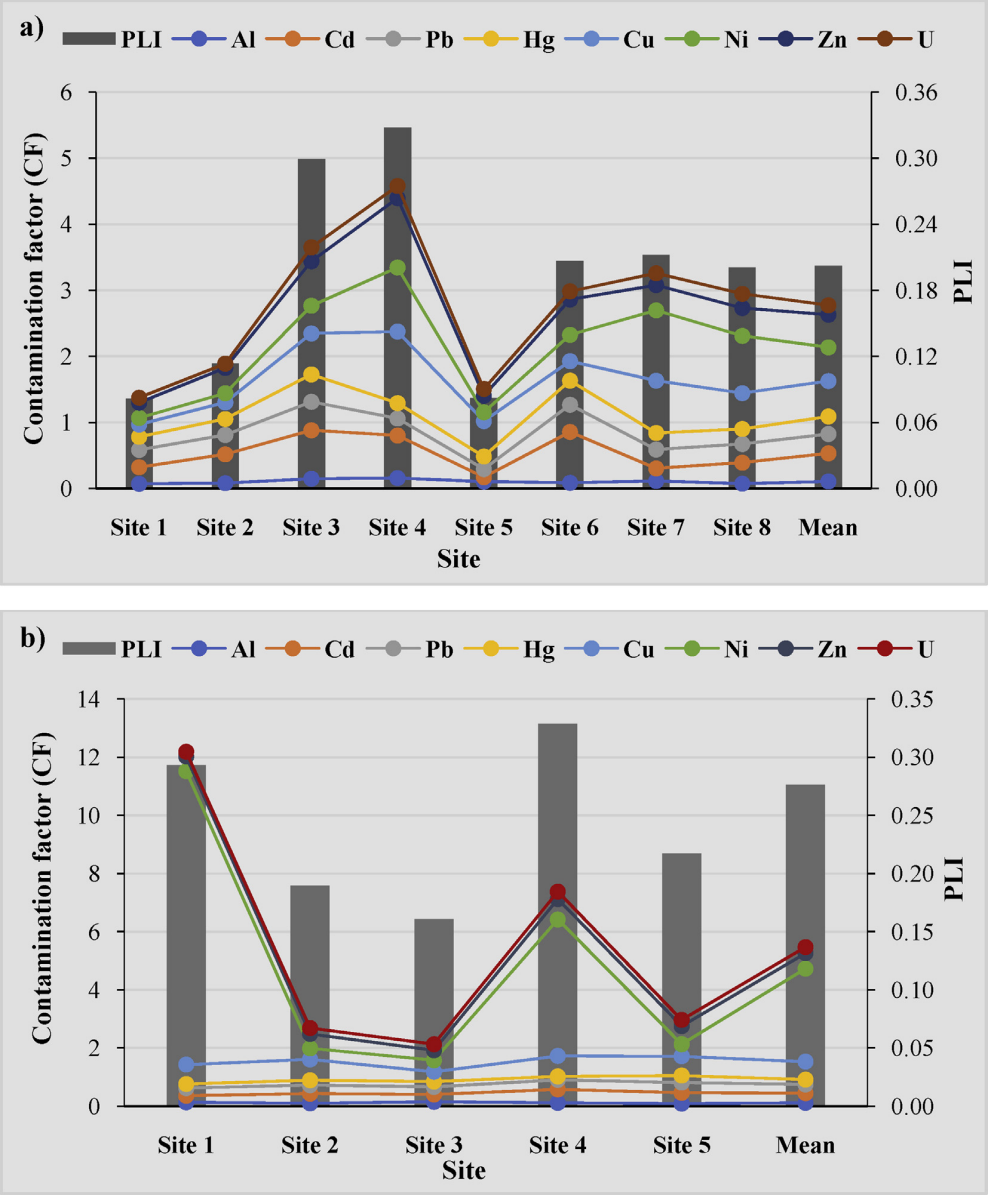
The contamination factor (CF) and pollution load index (PLI) of heavy metals in the sediments of the Abiete-Toko gold district: a) Kienké watershed; b) Tchangué watershed.

According to Hakanson's classification (1980), CF of all metals in the sediments of the Abiete-Toko watersheds showed low contamination factor in all selected sites. All the values are less than 1 (Table 6; Fig. 5a, b). These same values are observed in the pollution load index which is determined for contamination severity and its variation along the rivers. The values on PLI in this study are very low and considered as unpolluted (Table 6; Fig 5a, b). The sediments were lowly contaminated by theses metals due to the influence of external discrete sources like artisanal gold mining activities, agricultural runoff, and other anthropogenic inputs.

**Table 6:** The contamination factor (CF) and Pollution Load Index (PLI) of the Abiete-Toko watersheds.

	Site	Contamination factor (CF)								PLI
		Al	Cd	Pb	Hg	Cu	Ni	Zn	U	
Kienké watershed (Abiete)	Site 1	0.07	0.25	0.26	0.20	0.19	0.10	0.23	0.07	0.08
	Site 2	0.08	0.44	0.29	0.25	0.25	0.14	0.38	0.06	0.11
	Site 3	0.15	0.73	0.43	0.41	0.62	0.42	0.67	0.21	0.30
	Site 4	0.16	0.64	0.26	0.23	1.08	0.97	1.05	0.18	0.33
	Site 5	0.10	0.07	0.12	0.19	0.53	0.13	0.25	0.10	0.08
	Site 6	0.09	0.77	0.41	0.36	0.29	0.40	0.54	0.12	0.21
	Site 7	0.12	0.19	0.28	0.25	0.79	1.06	0.38	0.18	0.21
	Site 8	0.08	0.32	0.28	0.23	0.54	0.87	0.42	0.22	0.20
	Mean	0.11	0.43	0.29	0.27	0.54	0.51	0.49	0.14	0.20
Tchangué watershed (Toko)	Site 1	0.13	0.23	0.26	0.14	0.67	10.09	0.50	0.17	0.29
	Site 2	0.10	0.33	0.29	0.17	0.72	0.38	0.49	0.20	0.19
	Site 3	0.15	0.25	0.26	0.18	0.34	0.40	0.34	0.21	0.16
	Site 4	0.12	0.46	0.33	0.12	0.70	4.69	0.70	0.26	0.33
	Site 5	0.09	0.37	0.35	0.24	0.66	0.41	0.62	0.22	0.22
	Mean	0.12	0.33	0.30	0.17	0.62	3.20	0.53	0.21	0.28

### 3.3 Physical degradation of ecosystem

**Fig. 6:**
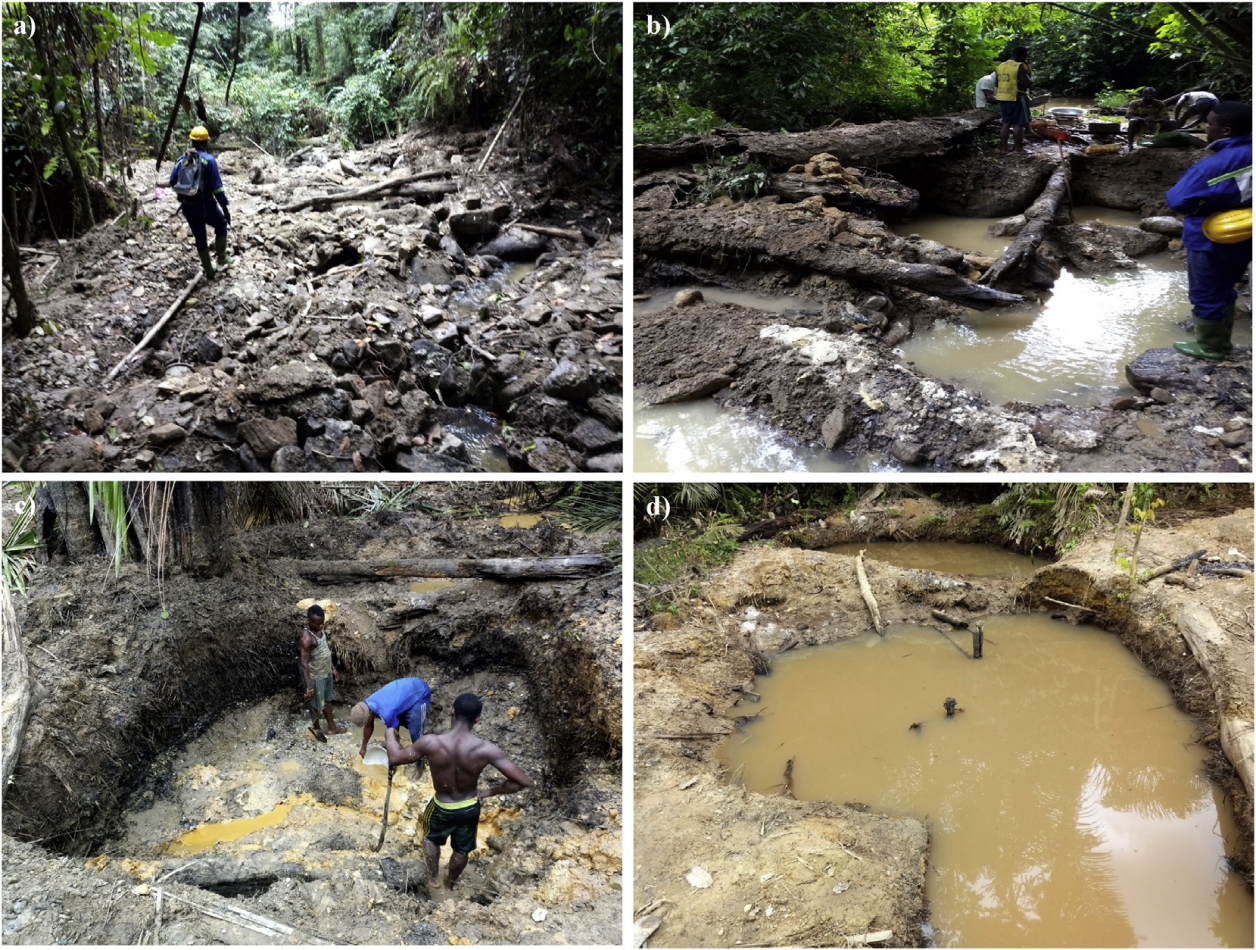
The physical degradation of ecosystems: a) destruction of the forest ecosystem; b) relocation of stream beds; c) land section with rectangular shape and d) geometric forms according to the layout of the mining grounds.

In the Abiete-Toko watersheds, disruption of the environment following mining activities leads to visible changes in the landscape (Fig. 6) and generates thousands of tons of tailings. Mining is therefore responsible for deforestation and soil destruction. To access the ore, the opening of access roads, the prior clearing of selected areas for camp development and the storage of equipment contribute to the destruction of the forest ecosystem and the relocation of stream beds of the watersheds (Fig. 6a, b). The construction of retaining walls and the satisfaction of current needs on the sites accentuate deforestation (Fig. 6a). Digging very often begins with the delimitation of a section of land, often rectangular in shape (Fig. 6c). In addition to this regularly used form, other geometric forms can be defined according to the layout of the mining grounds (Fig. 6d). At the end of gold panning work, these mining structures (usually more than 2 m deep) are subsequently abandoned without any rehabilitation and without being closed. All these activities in course of evolution lead to a gradual flora and fauna (main ecosystem constituents) destabilization.

## 4 Conclusion

The present study quantified and assessed the natural enrichment or anthropogenic sources, contamination levels and toxicity of some heavy metals and U in sediment samples from two watersheds of the Abiete-Toko gold complex. It shows that the distribution of these metals in sediments is not uniform over the watersheds and the change in concentration is due to the release of these metals from different anthropogenic sources. The Cu and Ni concentrations of site 4 of the Kienké watershed are higher than the standard average values. Whereas in Tchangué watershed, only Ni is higher than the standard average values. The site 4 of Kienké watershed and the Tchangué watershed are polluted by Ni and might create an adverse effect on the river's ecosystem. The EF values revealed that sediments in this investigation are moderately polluted by Ni and Cu and unpolluted by other metals with is confirmed by Geo-Accumulation Index. These sediments have significant to very high ecological risk assessment in Ni and Cu whereas the watersheds are generally unpolluted by trace metals and U. In this area, artisanal gold mining activities, agricultural runoff, lithology, and other anthropogenic inputs are probable sources of that slight metal pollution. This is due to the physical degradation of the ecosystem through excavations, wells and other stream excavation methods rapidly expanding in the area. Appropriate measures should be taken by local authorities (e.g. by creating fish ponds) to rehabilitate the gold mining sites, to ensure appropriate treatment of wastewater and non-use of toxic effluents into nearby tributaries.

## Declarations

### Author contribution statement

Eugène Pascal Binam Mandeng, Louise Marie Ngo Bidjeck Bondje: Conceived and designed the experiments; Performed the experiments; Contributed reagents, materials, analysis tools or data.

Armel Zacharie Ekoa Bessa: Conceived and designed the experiments; Performed the experiments; Analyzed and interpreted the data; Contributed reagents, materials, analysis tools or data; Wrote the paper.

Yvan Demonstel Ntomb, Jacques Wassouo Wadjou, Elvine Paternie Edjengte Doumo, Lucien Bitom Dieudonnè: Conceived and designed the experiments; Contributed reagents, materials, analysis tools or data.

### Funding statement

This research did not receive any specific grant from funding agencies in the public, commercial, or not-for-profit sectors.

### Competing interest statement

The authors declare no conflict of interest.

### Additional information

No additional information is available for this paper.
